# “There’s a lot of people who love them, so why call ‘em junkies?”: clinician and patient perspectives about words used to describe people who use drugs

**DOI:** 10.1186/s13722-025-00591-w

**Published:** 2025-09-02

**Authors:** Gayathri Sundaram, Taisuke Sato, Brindet Socrates, Alysse Wurcel

**Affiliations:** 1 Tufts University School of Medicine, Boston, MA USA; 2 Kansas City University, Kansas City, MO USA; 3 Tufts Medical Center, Boston, MA USA; 4 Boston Medical Center, Boston, MA USA; 5 Boston University School of Medicine, Boston, MA USA

**Keywords:** PWUD, Stigma, Person-first language, Qualitative research

## Abstract

**Background:**

There is increasing attention in clinician care to the importance of using person-first language. Clinicians’ words can reinforce clinicians’ pre-existing stigmas and biases. People who use drugs (PWUD) continue to face stigma from clinicians. Person-first language is a way to reduce stigma and perpetuation of bias.

**Methods:**

Through specific structured in-person interviews, we examined the usage of stigmatizing language in the care of PWUD by surveying key clinicians– such as physicians, nurses, and social workers—and patients who self-identified as PWUD at Tufts Medical Center (Boston, MA) between July 2022-September 2022. Interview guides were created using the Consolidated Framework for Implementation Research (CFIR) 2.0 as a framework. We evaluated perceptions of person-first language and barriers to using person-first language amongst participants. Interviews were coded with Dedoose Software and inductive thematic analysis (ITA) methods were used until all themes were captured; CFIR 2.0 determinants used during interview guide creation were used as preliminary themes and modified as needed.

**Results:**

We interviewed thirty-four people, including eleven PWUD at time of interview. Most clinicians agreed that language is important and matters when talking to patients and during documentation. Almost all patients agreed that language was important to them and impacted their relationship with their provider. However, there were responders that felt that person-first language was unnecessary, ineffective, and overly verbose in the medical setting. Major barriers to using person-first language were unawareness, lack of formal training, and perceived generational differences in appropriate language.

**Conclusion:**

Addressing language usage is a critical opportunity to promote inclusion and reduce bias amongst PWUD. As medical charts become increasingly accessible by patients, the use of language by the clinician becomes increasingly important. To create and maintain equitable systems of care, it is important to meet clinicians where they are at and to work with them to address these issues. This can include targeted educational sessions and resources informing clinicians on preferred language use and curriculum for providers-in-training.

**Supplementary Information:**

The online version contains supplementary material available at 10.1186/s13722-025-00591-w.

## Background

Stigma negatively impacts engagement of people who use drugs in healthcare. Substance use is prevalent in the United States, with 61.2 million people over 12 years of age using “illicit drugs” in 2021 [1]. In 2022, there were 107,941 drug-involved overdose deaths, driven mainly by synthetic opioids [2]. There are effective harm reduction tools for treating substance use disorder and infectious sequelae of substance use disorder, but people who use drugs may choose not to access or delay access to care because of stigma [1, 3]. PWUD develop strategies to avoid healthcare interactions, such as delaying presentation, downplaying pain, not disclosing drug use, and seeking other care [4, 5]. 

The choice of words used to describe PWUD and the process of using drugs is important. The stigma against drug use and PWUD continues to be manifested through the usage of words that are insensitive, exclusive, and dehumanizing, such as “junkie”, “addict”, or “drug abusers”. Stigmatizing language is a broad umbrella that includes terms like these, and one method to avoid stigmatizing language is through the use of person-first language. Person-first language allows for the separation of individuals and their disorder, disease, condition, or disability [6]. Person first language has been agreed upon as the preferred language for SUD by a number of organizations, as there has been a push to respect the dignity of PWUD [6–10]. Person-first language is important not only when communicating orally, but also in medical documentation. As medical documentation is often copied from prior descriptions in the electronic medical record, stigmatizing language used in one medical note often becomes a default attribute of an individual’s medical chart. Thus, the presence of insensitive language often persists and can cause unconscious bias among future clinicians reading a patient’s medical chart. Harmful words may also carry downstream effects on future healthcare providers. The language used in medical notes influences providers-in-training and their patient views as they learn from these notes. In turn, their professional behavior during patient encounters and in medication prescribing is negatively impacted [11]. When comparing “having a substance use disorder” and “substance abuser,” “substance abuser” was found to elicit and perpetuate stigmatizing attitudes amongst clinicians [12]. 

The language used by clinicians to discuss medical issues is often different than the language used by patients. While it is considered the standard of care to use person first language, it is unclear whether this is practiced often when it pertains to substance use, and by whom [6]. Additionally, while clinicians may be using this language, it is unknown what the perception of this language is by PWUD, whether they prefer it or have any preferences, or if the language used by their clinician has downstream effects on the patient-clinician relationship. Addiction care requires deep trust and cooperation between the patient and clinician. Recognizing the differences in how patients and clinicians discuss addiction, and the reasons behind them, can lead to more patient-centered care with reduced stigma. Based on our combined clinical experience, we identified disjointed perspectives between patients and clinicians in the relative importance of language in the context of healthcare as well as understanding the variability in patient perspectives. In this study, we used qualitative methods to analyze patient and clinician perspectives on person-first language to describe PWUD.

## Methods

### Setting and participants

The project was conducted at Tufts Medical Center, a tertiary not-for-profit academic medical center located in Boston, Massachusetts between July 2023 and August 2023. Patients admitted to the Tufts Medical Center medical units interact with many members of the hospital care team, including nurses, residents, attending physicians, caseworkers, and pharmacists. The goal of this study was to recruit patients with substance use and a representative sample of the care team they might interact with in the hospital. Patients were referred to the study by residents and attendings on various inpatient services if they met inclusion criteria (spoke English, at least 18 years of age and reported current or past drug use within one year of opiates, opioids, cocaine, or methamphetamines as reported by the primary hospital team). Inclusion criteria for clinicians included if they were employed at Tufts Medical Center and if they interacted with patients who identified as PWUD in their clinical practice directly or indirectly. Clinicians were identified using purposive sampling by AGW, a member of the Tufts Medical Center Opioid Task Force, who has a list of member emails; these members are clinicians known to be involved in the care of PWUD. Members were emailed with an introduction to the study and requested (1) their participation and (2) referral to other clinicians who should be interviewed who meet inclusion; the recruitment pool thus included clinicians who were part of the Opioid Task Force and those who were not. The research team (GS and TS) emphasized to participants that participation in the study was voluntary, and no personal identifiers would be attached to their responses. Interviews were conducted in-person in various units and bedside in hospital, and verbal consent was gathered. All study protocols were separately reviewed and approved by the Tufts University Health Sciences Institutional Review Board.

### Study tool development

Using the implementation science framework, person-first language is considered the evidence-based intervention. Two in-depth interview guides were created, one for PWUD and one for clinicians using the Consolidated Framework for Implementation Research (CFIR) 2.0 [13]. As person first language is the recommended method of communication in healthcare, CFIR 2.0 was applied as it is a multilevel approach to identify factors influencing the implementation of practices through analysis on various levels, such as individuals, organizational context, and the implementation process itself. CFIR-based interview is best suited when identifying factors facilitating or hindering changes in healthcare, as it is an entrusted framework designed with the dynamic nature of healthcare organization in mind. The CFIR 2.0 additionally considers how constructs interact with each other, which is well suited for this study, The CFIR 2.0 provided a framework for each interview guide in which questions were created based domains such as the Inner Setting Domain, Individuals Domain, and the Characteristics Domain. These domains along with the constructs used to develop the interview guide questions were then used later in data analysis as preliminary codes. In the clinician interview guide we had questions about clinicians’ feelings about providing care to PWUD and in the interview guide for PWUD, we asked questions about experiences in healthcare settings. For both groups, familiarity, usage, and perception of person-first language was also assessed. We asked patients and clinicians to reflect on data from a publication showing that person first language improved hospitalization outcomes for PWUD [14]. Some examples of questions for clinicians included “There’s a lot of words that can be used to talk about drug use. Could you list some words that you have heard being used?” and “How has language on drug use changed?”. Similar questions that were asked to PWUD included “Tell me about the language people in the hospital use to talk about drug use.” and “Does it matter how clinicians talk about you?” (See supplemental materials for full interview guides).

### Data collection and analysis

Interviews were conducted in-person and audio-recorded; no notes were taken during interviews. The research team (GS and TS) reviewed consent forms orally and obtained oral consent before administering the interview; regarding credentials, both members of the research team have bachelor’s degrees, primarily worked as research assistants during this study, and were both trained by principal investigator (AGW) to conduct these interviews. The research team did not have a relationship with any of the participants prior to interview, and participants were informed that the research team was conducting this study to understand the types of language used in the hospital to talk about drug use and the people who use them. Interview guides were pilot tested among other members of the research staff that were not involved in this study. The research team did not deviate from the written script unless to provide definitions or minor clarifications to avoid biasing participant responses. All responses were stored under unique participant-specific codes to maintain anonymity. Demographic information was collected at the end of each interview. Interviews lasted between 25 and 35 min and were conducted in either the clinic or hospital room for patients and in the office setting for clinicians; no one else was present during interviews besides the interviewer and interviewee. Patients received reusable insulated water bottles since monetary compensation was not permitted, and clinicians received a $20 electronic gift card upon completion of the interview. No repeat interviews were conducted. Audio files were transcribed using a professional transcription service (Landmark Associates, Inc.) and coded in Dedoose [15, 16]. Transcripts were not returned to participants for comment or revision, and incorrect transcriptions were at the discretion of the research team. The research team created a preliminary deductive codebook and coded a subsample of interviews. The preliminary deductive codebook was based on the CFIR determinants used to create the interview guides. These topics were used by the research team to independently code the same four interviews, two patient interviews and two clinician interviews. After coding independently, the research team regrouped and analyzed this preliminary data to consolidate the identified codes and eliminate redundancies to create a preliminary code book. This was repeated for two more patient and two more clinician interviews to identify any new codes. With this preliminary code book, the research team independently coded the remaining interviews. Any themes that did not correlate to CFIR 2.0- related domains were added organically by each member of the research team independently, using inductive thematic analysis (ITA). Coding was developed iteratively until thematic saturation was reached and all themes were captured. Upon completion of coding, the research team reconvened and consolidated similar codes as appropriate. Groups of codes were then further grouped under larger themes, with a final number of 131 codes.

## Results

Fifty-nine clinicians were approached through email or in-person discussions and twenty-three consented to participate in the study. Of these twenty-three clinicians, nine were clinicians in internal medicine, internal medicine subspecialities, or emergency medicine; five were either clinicians in intensive care or surgical specialties; three were members of the pharmacy and laboratory departments; four were in nursing; two were clinicians in case management and physical therapy. Ten of the clinicians interviewed were attendings, two were fellows, one was a resident, and one was a physician assistant. Eleven patients who self-identified as PWUD were also recruited and interviewed. The demographics of the cohort can be found in Table 1.

**Table 1:** Demographics of study participants

	Patients identifying as People Who Use Drugs (PWUD)	Clinicians
**Number of Participants**	11	23
**Age**		
**Average**	51.27	43.39
**Median**	56	41
**Range**	23	39
**Gender**		
**Men**	7 (64%)	13 (57%)
**Women**	4 (36%)	10 (43%)
**Additional genders**	0 (0%)	0 (0%)
**Race**		
**White**	4 (36%)	11 (48%)
**Black**	1 (9%)	4 (17%)
**Asian/Pacific Islander**	0 (0%)	6 (26%)
**Native American**	1 (9%)	0 (0%)
**Other**	4 (36%)	0 (0%)
**Unknown**	1 (9%)	0 (0%)
**Asian/Pacific Islander**,** Other**	0 (%)	1 (4%)
**White**,** Other**	0 (0%)	1 (4%)
**Ethnicity**		
**Hispanic**	4 (36%)	0 (0%)
**Non-Hispanic**	7 (64%)	23 (100%)
**Highest Level of Education**		
**Less than High School**	1 (9%)	0 (0%)
**High School/GED**	5 (45%)	0 (0%)
**Some College**	4 (36%)	0 (0%)
**Associate’s Degree**	1 (9%)	0 (0%)
**Bachelor’s Degree**	0 (0%)	2 (9%)
**Graduate Degree**	0 (0%)	4 (17%)
**Doctoral Degree**	0 (0%)	17 (74%)

### Near universal agreement about the negative impact of using dehumanizing words

Patients reported negative feelings associated with the word addict, even when used in traditional recovery situations:

They’re loser. They’re no good. They’re useless people.’ That comes along with that stigma right there. That’s why I hate that word. That’s why even when I go to NA meetings, I hate to say, when they say, raise your hand, say, ‘I’m an addict,’ I won’t do that. I don’t even like to admit to I’m an addict because it still has a stigma to it. Even though that I’m in a meeting, they don’t look at you like that, but it’s something about psychological that I’mma keep telling myself, ‘I’m an addict. I’m an addict. I’m an addict.’ ‘No, no, I’m not an addict. No, I’m in recovery.’ I say.”

The term “addict” made patients feel like they were placed “in a box” and dehumanized. One person said felt that providers do not understand them and what the term “addict” means to them:

Addict, a lot of people don’t understand the word addict. They just see somebody that’s on Mass Ave [an intersection in Boston, MA with visible drug use], or they think of something that people who really—that that’s what an addict is, but that’s not really what an addict is. Addict could be any one of us. It could be a person could be working with you besides you right now, and you could be addicted to something, and you don’t even know it.

Some felt it was acceptable to identify and call themselves an addict but preferred their healthcare team did not: “I mean, I don’t like being called an addict. I think that when I call myself an addict, it’s fine.” Others had no preference on what words were used to describe them. “Yeah, I’m a user, I’m an addict, so what? That’s it. That’s it and we’re not thinking about.” Another word reflecting de-humanizing language that emerged in interviews was “junkie.” One patient said “somebody loves that person. You know what I mean? No matter what they are, no matter what they’ve done… There’s a lot of people who love them, so why call ‘em junkies?”

Overall, the term “addict” held different values to different PWUD interviewed as some self-identified and called themselves an “addict” but clarified that it was acceptable only for them to say that about themselves; they did not appreciate hearing others refer to them as an addict. Some felt that addict was not the worst term in comparison to other terms they had heard, such as junkie, and thus preferred addict over the alternatives. In some cases, context mattered for the term addict, as “addicted to” was preferred over addict, possibly due to this statement being more accurate to the individual’s current state while acknowledging their prior drug use. There was a strong desire for plain, simple language: calling patients by their name, saying that they are a PWUD, and seeing and respecting them as people.

Clinicians also clearly felt that terms like “addict” and “junkie” were not correct. One case manager said, “[we] should have enough professionalism to say, ‘We don’t use that term anymore,’ cause we can’t rely on, ‘Well, that’s how I grew up,’ or, ‘That’s what they said when I was a teenager.’” A nephrology fellow said, “[I] personally have witnessed a difference and have had individuals who use drugs who I have cared for in the hospital share their experiences with me in the way that they’ve been spoken to and spoken about and how it affects their self-esteem and their forthcomingness about their drug use and their just general comfort interest with the medical system. Absolutely, I think that language matters.” Other clinicians mentioned using formalized language in charts, with a rheumatology attending physician stating: “I think many of us have paid extra attention to try and not use language that can be interpreted as offensive in our notes because we know that patients read them.”

### Most patients feel Person-First Language supports their relationship with clinicians

When asked if it mattered how clinicians talked about them, one patient said “it does matter a lot because it shows me that you care… if you don’t care, I’ll walk right out. I’ll just walk out, and I’ll go find somebody else that will give me the care and understanding that I need when it comes to my healthcare. I definitely will do that.” Another patient said “talk to me with respect, and me and you can have a conversation. Now if you come to me and say something to me that I feel disrespected, I won’t deal with you.” The theme of respect was mentioned again as a patient brought up how feeling respected by their care team allowed them to “be honest with ‘em and tell ‘am what’s really going on with me… whatever I’m going through, they’ll build something around that, so that way I can get support to all the help I need.” One patient reflected on the term “addict” made them feel, “[I am] embarrassed about it. I feel ashamed…I feel like I’m nobody.” Another patient said that “it’s important that they respect us and that they talk about us like we’re human beings. Not like we’re just some losers on the street. We’re still somebody’s son or daughter or mother or father or brother or sister, or friend.”

### Most clinicians support Person- FirstLanguage, but some feel it is complex and unnecessary

A patient described person-first language as “a little spin on words, but I think it’s almost like a cognitive thing. Like where it almost teaches you in a subtle way to—it’s just a more positive way to say it…. If you say it like that on a regular basis, it probably just makes you feel better about yourself over time. I get that.” Some attendings agreed with the change in cognition that person-first language allows. A cardiology attending physician explained the value of person first language as reinforcing the concept that addiction is a disease: “Well, it used to be a very stigmatized failure of personal morals or failure of willpower or a general weakness in character. Now I think that the contemporary view is that well, it’s a disease addiction like any other disease, and we have to treat it as a disease rather than as a moral failing.” A neonatology attending physician described how person first language reinforces respect for PWUD: “I think you’re getting close to the underlying belief of respecting human beings, no matter what age, what background they were, educational or socioeconomic background, I think a person is a person. There are a lot of conditions that it’s unfortunate, right, that happens to many of us. I really hope that the next few years, I will continue to hear, person first language.” A subset of clinicians and healthcare workers agreed that addict was a judgmental term preventing patient growth, and could appreciate the usage of person first language to make the health concern of drug use more medicalized while showing appropriate respect to their patients.

A perceived complexity to using person first language to many was lack of familiarity and comfort with terms, potentially due to a generational divide: “My old man reaction is ‘oh, not again, not more of this,’” “I graduated from medical school ten years ago. It’s the vernacular for junior faculty, it’s not the vernacular for the older attendings… I think it’s harder for people to change.” There was a subset of clinicians who saw the value of person first language but felt that it was not conducive for medical purposes due to length, as a physician assistant noted that they “work with people with remarkably short attention spans who like to use very direct stuff… it feels a little bit more like a semantics issue.” An intensive care attending physician said “I know that we’re trying to get away from drug abuse and using abuse for all sorts of stuff, but I still call it that. I find a lot of—I consider myself a tolerant person, but I consider a lot of the work around the linguistics of marginalized populations—I spend more time thinking about using the right word than taking care of the patient. That is where I’ve had to just default to the language I was raised with, which is drug abuse… I appreciate the intention behind changing of language and word use, but I think sometimes people are trapped in the way that they learned how to describe things and the intention behind not using the updated terms is not always malignant.” The “wordiness” of person-first language was seen as counterintuitive to the emphasis of being succinct in medical contexts, with some concern that using person first language may be performative rather than impactful. A portion of clinicians interviewed argued that this was a case of modernizing medicine to become “politically correct.”

Both clinicians and patients felt that changes in words did not matter as much as action. One patient stated “It’s the emotions and expressions, the actions… you can see the expressions like, ‘Oh, my God, here’s another loser. Here’s another person that don’t need no help.’” Another patient noted, “I really can’t say that I’ve ever heard any of the doctors and nurses really talking about [my drug use] in front of me. I just really seen the behavior and the judgment, and the way they act. You kinda just get that vibe around the way they treat you, and the attitudes and the behaviors… You just can feel it.”

An intensive care attending physician said “I think words, for me, are less important than how you treat people. If you look at things in general to say, “Oh, drug abuse is worse than people who use drugs,“—and I don’t even know what the other, most updated terms are… I get the point of it.” When asked if person first language mattered, a nephrology attending physician said “For me, no. For society, perhaps.” In response to the data a recent publication’s finding of an association between treatment for opioid use disorder prescriptions and usage of person first language, there were a subset of clinicians who doubted the data: “Providers using person-first language are most likely to prescribe methadone, suboxone? I don’t know. I mean I don’t think it—I don’t know that it would change my approach to things… I would be curious to know where that study was done, and what kind of environment that it was done in, and who the prescribers are because I don’t know that that would change the way that I—I manage the population the same regardless of how we name them, I guess.” Overall, clinicians had varying views on language, ranging from being in complete support of person-first language to believing that language is irrelevant if patient treatment is unbiased.

### Clinicians reported lack of training for Person- First Language

There appeared to be some shortcomings regarding clinicians having sufficient knowledge about person first language. Many expressed that person first language is a recent idea and thus it is being used more naturally by recently trained clinicians: “as I said, people who are either in training, in medical school, or recent graduates there’s been a big push to use person-centered or person-first language as opposed to people who completed their training many years ago. I think these concepts either have not been introduced to them, maybe haven’t been introduced, maybe haven’t been reinforced, and, therefore, they’re less likely to use.” A nephrology attending physician felt that “younger people, medical students and interns and residents are more likely to use it.”

There was emphasis on lack of training: “the language I was raised with, and my training, is what comes out,” “I don’t think people are trained to use it,” “No one’s ever really trained me.” A medical laboratory director felt that lack of awareness resulted in lack of implementation of person first language: “I think the problem is that, unless you’re well aware of it and you use it all the time, it’s just not going to come up… It can be an easy thing to just slip your mind because it’s not necessarily the way in which we formulate sentences and statements as we think and as we speak.” A physical therapist saw person first language as “a learning curve that we have to get over.” Many felt overwhelmed by the choices of language available, not knowing which language was appropriate, and feeling unsure about whether patients held a strong preference for their language use.

## Discussion

**Fig. 1:**
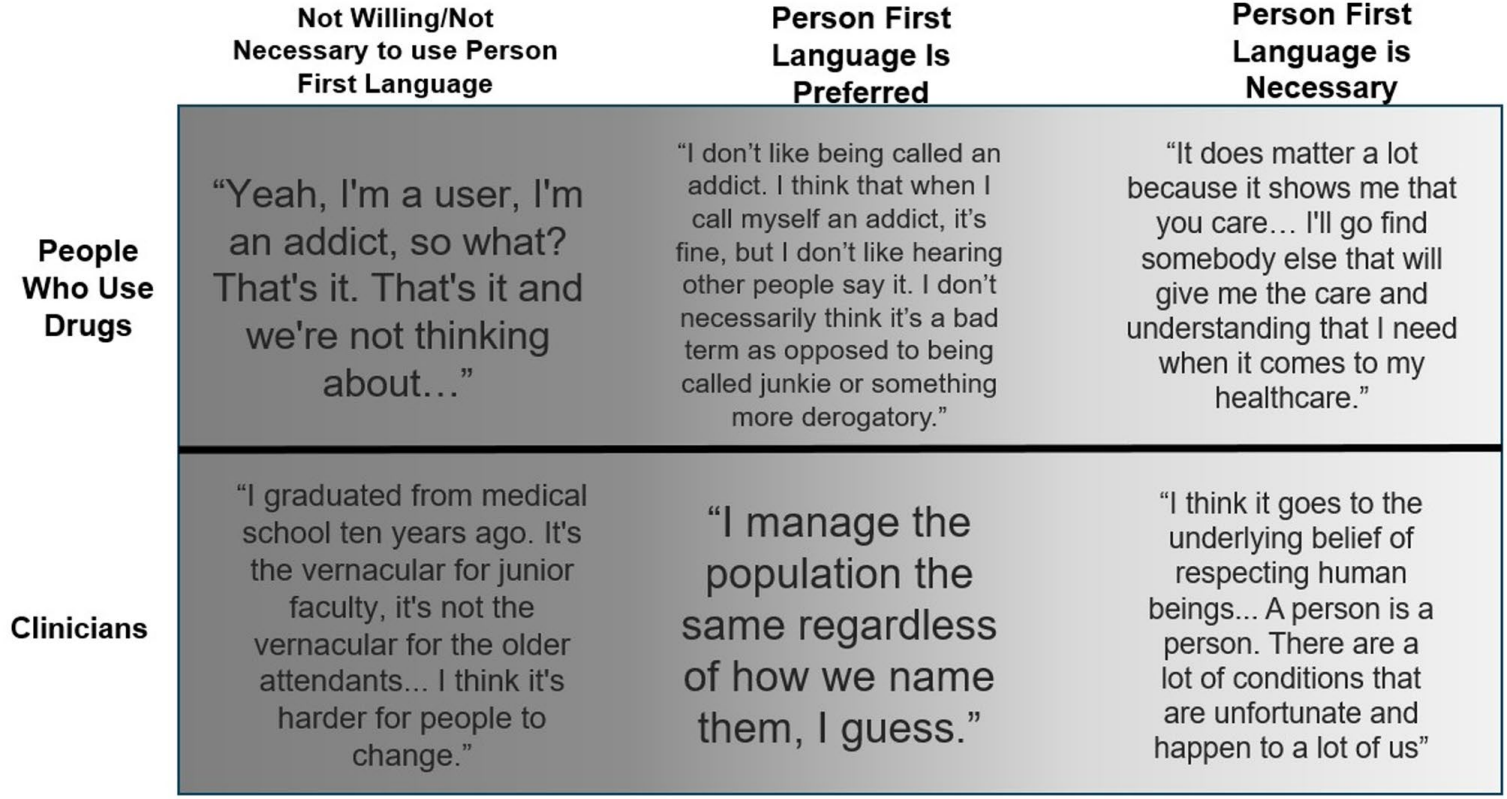
Select quotations from participant interviews

We report on areas of consensus and divergences within and between patients and clinicians about the importance of person first language. While most clinicians were able to appreciate the value of person first language, some were vocal about their disbelief in its potential impact and relevance in the medical setting. Misconceptions such as complexity and difficulty of use hindered the usage of first-person language among clinicians surveyed. There was a strong preference among patients for providers to use language that demonstrates respect for their humanity and medical conditions and, when this language was used, they reported improved clinician-patient rapport. A visual representation of the range of responses from patients and clinician can be seen in Figure 1.

Stigmatizing language impedes equitable healthcare. It was found that some providers were unconvinced by the impact that person-first language has, or that the language used matters if the treatment is standard level of care. This is contrasted by findings of prior studies that have shown how negative associations do exist when using stigmatizing language, verbal or written [12, 17–21]. Some findings of this study were the overwhelming desire to be treated with respect and the usage of plain, simple language in reference to their drug use, while being able to refer to oneself as an “addict”. Other research has noted similar findings, with one study conducted at outpatient methadone treatment programs finding a preference for providers to use “person with an addiction” and “person with substance use disorder,” and another study observing that preferences by individuals who use heroin and who are in early recovery are consistent with person-first language guidelines despite some using the term “addict” to refer to themselves [22, 23]. The term “addict” has been found in another study to hold a higher level of negative association compared to “person with a substance use disorder,” however “person with a substance use disorder” was still found to hold negative associations; this was reflected in our own findings of PWUD’s desire for plain language to describe them [24]. High stigma levels have been associated with less open communication with medical providers, and many PWUD noted that they felt hesitant to share their drug use out of fears of judgment; it has been found that people who inject drugs (PWID) with high levels of enacted stigma were less likely to seek treatment for hepatitis C (HCV) [3]. Additionally, due to anticipation of stigma, some PWID develop strategies to avoid healthcare interactions, such as delaying presentation, downplaying pain, not disclosing drug use, and seeking other care [4, 5]. Negative words promote feelings of shame, hopelessness, and isolation by implying an individual’s identity is equivalent to the medical condition they are facing. Our findings reinforce the concept that the words that PWUD hear can impact how they view themselves and their drug use and impact their relationships with their healthcare team.

The lack of information about person-first language, either due to generational disconnect or due to lack of training, was a perceived barrier in clinician usage. Generational differences in the practice of medicine may be influencing the perception of the impact of language, as prior research has shown that clinicians licensed after 2000 were more likely to make changes in documentation once notes became open to patients than those licensed before 2000, with the changes most frequently being related to the use of language that could be perceived as critical of the patient [25]. Person-first language implementations could be supported through early education in medical training, discussions in medical team settings on appropriate language use, and open forum sessions in which providers can openly discuss their hesitancy in implementing person first language. Many PWUD not only expressed but emphasized that addiction is a disease and should be treated as such. First-person language is the right step in achieving the change in perception. Programs have been implemented at a medical education level and have been shown to improve provider comfort and understanding of SUD, with one study demonstrating increased confidence in residents in encountering SUD in clinical practice after a 2-part curriculum [26]. Another study conducted amongst medical students found a significant reduction in surveyed stigma towards PWID amongst first year medical students after a mandatory 1.5-hour session on PWID care [27]. These studies are promising indications that focused educational discussions have the potential to correct misconceptions and educate providers about care of PWUD, as our findings noted a general theme in the lack of knowledge base and training in what was “right” to say. Additionally, it is important to keep PWUD involved in conversations about language used surrounding addiction care when educating clinicians; while it should be done in a way that is respectful of these individuals’ lived experiences, co-teaching about substance use has been shown to be meaningful in shifting attitudes about the care of these populations [28]. 

There may be clinician and societal pressure to increase the use of person-first language. As medical charts become “open”, the use of language by the clinician becomes increasingly important. Stigmatizing language in a note could lead to negative health ramifications, such as distrust of medical systems and reluctance to seek care. Patients who experienced racial discrimination in a healthcare setting were less likely to follow their provider’s recommendations, less likely to receive recommended chronic disease screening, and more likely to delay care [29]. Our findings support this, with individuals noting how they felt less willing to open up to a provider who disrespected them.

There are limitations in this research study. Patient interviewees were identified and enrolled based on availability and reporting from clinicians; thus, this sample may not be proportionally representative of all patients with addiction that the medical center takes care of. In particular, self-selection bias is a concern, as consenting to a lengthy recorded interview about a sensitive topic such as addiction demonstrates increased willingness to engage with clinicians and researchers. Additionally, clinicians interviewed were initially recruited through the Tufts Medical Center Opioid Task Force member list. This may have caused a selection bias and a certain level of homogeneity in opinion and additional awareness of addiction medicine’s stigma, skewing them towards being more open and/or familiar with person-first language. Social desirability bias may play a role in participant responses.

Hearing directly from clinicians and patients is a necessary and meaningful step forward towards understanding how we should talk about addiction in healthcare and how we can actively reduce stigma that may discourage individuals from seeking medical help.

## Conclusions

Appropriate language use varies amongst clinicians, with major barriers to using person-first language consisting of lack of knowledge and familiarity. PWUD overwhelmingly wanted the language used by providers to be respectful of them and their substance use. Addressing language usage is a critical opportunity to promote inclusion and reduce bias amongst PWUD. As medical charts become increasingly accessible to patients, the use of language by the clinician becomes increasingly important. To create and maintain equitable systems of care, it is important to meet clinicians where they are at and to work with them to address these issues.

## Supplementary Information

Below is the link to the electronic supplementary material.

Supplementary Material 1

Supplementary Material 2

## Data Availability

The datasets generated and analyzed during the current study are not publicly available to protect participant privacy due to the sensitive nature of interview contents.

## Abbreviations

HCV: Hepatitis C virus
PWUD: People who use drugs
PWID: People who inject drugs
SUD: Substance use disorder
AGW: Dr. Alysse Gail Wurcel
GS: Gayathri Sundaram
TS: Taisuke Sato
