# Supplementary material for: “There’s a lot of people who love them, so why call ‘em junkies?”: clinician and patient perspectives about words used to describe people who use drugs
Source: Addict Sci Clin Pract. 2025 Sep 2;20:71. doi: 10.1186/s13722-025-00591-w (PMC12403917; doi:10.1186/s13722-025-00591-w)
Supplement: Supplementary file 1 — Supplementary Material 1 [file 13722_2025_591_MOESM1_ESM.docx]

**Provider Interview Guide**

“I have started the recording. You have agreed to being recorded and to participate in this discussion. However, you can skip questions at any time or stop participating if you want.

In this interview, we’d like you to comment on a range of patients that you’ve worked with and taken care of. However, if you would find it helpful to describe a specific situation, please feel free to give an illustrative example without using patient identifiers such as names and date of birth.

We will discuss the identification of patients with substance use disorder and the language used to discuss them in medical documentation.

| Question | Probe |
| --- | --- |
| What is your role in caring for patients? | What about patients with substance use disorder? |
| **Language Use** | |
| How do you ask people about drug use? | How do you feel about asking this question? Do you ask every patient? |
| What are some of the ways in which you identify a patient as someone who uses drugs? | - Are there other methods that you use to determine their drug use? - Do you use past medical records? |
| There’s a lot of words that can be used to talk about drug use. Could you list some words that you have heard being used? | What words do you use?  What words do you hear other people use?  Could you rank/order these words from best to worst? |
| Are the words you hear people use to talk about drug use different from the words used in charts? | How so? |
| How has language on drug use changed? | How do you feel about these changes? |
| **EMR**: Let’s switch to talking about the electronic medical record (EMR). The EMR can be used for clinical care, insurance billing, and research. | |
| How do you document drug use in your patients? | Do you write about a patient’s drug use in their medical record? What types of words do you use to describe this?  How has increased patient access to charts impacted the words you use? |
| If you wanted to see if a patient uses drugs, where would you look in the EMR? | If you were researching this, where in the EMR would you look? |
| Urine toxicology is used to identify people who use drugs in clinical and research settings. What do you think about this process? | What are some of the benefits? What are some of the downsides? |
| How often do you look to see if a patient you are admitted had a urine drug screen in the emergency room? | Have you ever ordered a urine drug screen on someone who was admitted? |
| Medications to treat opioid use disorder being present on the medical record is used to identify people who use drugs in clinical and research settings. What do you think about this process? | What are some of the benefits? What are some of the downsides? If someone presents on methadone, is that added to their medical record? |
| One method of identifying people who use drugs in research is through making a list of people who are on medications to treat opioid use disorder. What do you think about this process? | What are some of the benefits? What are some of the downsides? |
| **Transition statement**: Let’s come back to language use. | |
| Tell me what you know about person-first language. | An example is using person who injects drugs (PWID) instead of drug user. |
| How often do you hear people using this language? | What impact do you think person-first language for PWUD has? |
| On a scale of 1-10, with 1 being never and 10 being always, how often do you use person-first language? | What are some of the barriers against using person-first language? |
| There are some studies that show person-first language linked to medications for opioid use disorder (MOUD) treatment.  What do you think about this? |  |
| Do you think language about drug use matters? |  |

Lastly, I would like to ask you some demographics questions.

Age: _____

Gender: Male Female Prefer not to answer Other:_____________

Race: White Black Asian/Pacific Islander Native American Other Unknown

Ethnicity: Hispanic Non-Hispanic Unknown

If more than one race, circle all that apply.

What is your highest level of education: ___________________________

That concludes the interview. Thanks very much for your time and for the input you provided. If, after we leave, you have questions that arise, my contact information is listed at the bottom of the information sheet.”
