# Supplementary material for: “There’s a lot of people who love them, so why call ‘em junkies?”: clinician and patient perspectives about words used to describe people who use drugs
Source: Addict Sci Clin Pract. 2025 Sep 2;20:71. doi: 10.1186/s13722-025-00591-w (PMC12403917; doi:10.1186/s13722-025-00591-w)
Supplement: Supplementary file 2 — Supplementary Material 2 [file 13722_2025_591_MOESM2_ESM.docx]

**Patient Interview Guide v5 7.10.23**

“I have started the recording. You have agreed to being recorded and to participate in this discussion. However, you can skip questions at any time or stop participating if you want.

In this interview, we’d like you to comment on your own experiences in the hospital and your past or current drug use. Please feel free to share as much information as you are comfortably willing to share. As a reminder, this information will not be linked to your name or any personal identifiers. If you want to withdraw at any point, please let us know and we’ll stop the interview.

ICEBREAKER:

Just so I can get to you know you better, what is your favorite ice cream flavor?

| Question | Probe |
| --- | --- |
| How are you feeling about being at the (hospital/clinic) today? |  |
| Tell me about how your experience has been so far (at the hospital/in the clinic) | Is your experience here similar or different than previous healthcare experiences? |
| Tell me about how people who inject drugs are treated while they’re in the hospital/clinic | How do you feel like people are treating you in the hospital/clinic? |
| Tell me about how comfortable you feel discussing pain with your care team |  |
| Tell me about how comfortable you feel discussing withdrawal symptoms with your care team? | Tell me about how comfortable you feel discussing cravings with your care team? |
| Tell me about how it feels when a clinicians asks for a urine sample for drug testing. | Do you think urine drug tests are good tests to show what you are using? |
| There’s a lot of words that can be used to talk about drug use and people who use drugs. Could you list some that you know? | How do you feel about these words? Are there words you prefer?  Tell me about the words you hear other people use to talk about drug use. |
| Do you think that different people in the hospital use different words to talk about drug use or people who use drugs? | For example, nurses and doctors? Case workers? Social workers? |
| Has anyone spoken to you about your drug use while in the hospital? | What did they say? How did it make you feel? |
| Tell me about the language people in the hospital use to talk about drug use. | Is it positive or negative? Does it make you feel differently about your drug use or yourself? |
| Have you ever read any of your clinical notes? | Y🡪 How did it make you feel?  N🡪 Would you like to read your notes if you haven’t? |
| What words would you want clinicians to use to describe you? | For example, do you want them to say “someone who uses drugs?” |
| What do you feel about the word “addict?” |  |
| What words should researchers use to talk about people like you? | What words should researchers NOT use to talk about people like you? |
| Have you heard about person-first language? | Y🡪Tell me what you know about this. Where did you learn about this?  N🡪For example, people who inject drugs is a person-first language instead of “injection drug user” |
| How often do you hear people use person first language when talking about people who use drugs? | How often do they use this compared to people with cancer, people with diabetes, or other diseases? |
| Does it matter how clinicians talk about you? | What words do you want to hear them say? |
| Do you think the words clinicians use are related to the quality of healthcare? | Does language matter? |
| Do you think addiction is a disease? |  |
| Is there anything else you would like to share with us today? |  |

Lastly, I would like to ask you some demographics questions.

Age: _____

Gender: Male Female Prefer not to answer Other:_____________

Race: White Black Asian/Pacific Islander Native American Other Unknown

Ethnicity: Hispanic Non-Hispanic Unknown

If more than one race, circle all that apply.

What is your highest level of education: ___________________________
